# Tuberculous Granuloma: Emerging Insights From Proteomics and Metabolomics

**DOI:** 10.3389/fneur.2022.804838

**Published:** 2022-03-21

**Authors:** Abisola Regina Sholeye, Aurelia A. Williams, Du Toit Loots, A. Marceline Tutu van Furth, Martijn van der Kuip, Shayne Mason

**Affiliations:** ^1^Department of Biochemistry, Human Metabolomics, Faculty of Natural and Agricultural Sciences, North-West University, Potchefstroom, South Africa; ^2^Department of Pediatric Infectious Diseases and Immunology, Pediatric Infectious Diseases and Immunology, Amsterdam University Medical Center, Emma Children's Hospital, Amsterdam, Netherlands

**Keywords:** tuberculosis (TB), tuberculous meningitis, innate immunity, adaptive immunity, tuberculous granuloma, metabolomics, proteomics, biomarkers

## Abstract

*Mycobacterium tuberculosis* infection, which claims hundreds of thousands of lives each year, is typically characterized by the formation of tuberculous granulomas — the histopathological hallmark of tuberculosis (TB). Our knowledge of granulomas, which comprise a biologically diverse body of pro- and anti-inflammatory cells from the host immune responses, is based mainly upon examination of lungs, in both human and animal studies, but little on their counterparts from other organs of the TB patient such as the brain. The biological heterogeneity of TB granulomas has led to their diverse, relatively uncoordinated, categorization, which is summarized here. However, there is a pressing need to elucidate more fully the phenotype of the granulomas from infected patients. Newly emerging studies at the protein (proteomics) and metabolite (metabolomics) levels have the potential to achieve this. In this review we summarize the diverse nature of TB granulomas based upon the literature, and amplify these accounts by reporting on the relatively few, emerging proteomics and metabolomics studies on TB granulomas. Metabolites (for example, trimethylamine-oxide) and proteins (such as the peptide PKAp) associated with TB granulomas, and knowledge of their localizations, help us to understand the resultant phenotype. Nevertheless, more multidisciplinary ‘omics studies, especially in human subjects, are required to contribute toward ushering in a new era of understanding of TB granulomas – both at the site of infection, and on a systemic level.

## Introduction

Tuberculosis (TB) is a devastating infectious disease of pandemic proportions, caused by the bacterium *Mycobacterium tuberculosis* (*Mtb*). For many years, it has been one of the top ten causes of death from a single infectious agent (1). Although there has been a decline in the number of TB cases in some geographical regions, the rise in drug-resistant TB poses new challenges (2). TB is mostly a pulmonary disease, although it can affect other organs of the body, in which case it is known as extra-pulmonary TB (EPTB). The most severe form of EPTB occurs in the central nervous system (CNS-TB), with a prevalence of approximately 1% of the total TB cases reported (3), leaving about half of its patients either dead or permanently neurologically disabled (4).

Granulomas are the body's response to chronic antigenic stimulants. They represent the body's first line of response to *Mtb* infection and thus serve as a histopathological hallmark of TB. They comprise a combination of specialized immune cells influenced by an orchestrated pro- and anti-inflammatory response (5, 6). Granuloma formation can be affected by the number and location of these cells or other underlying diseases in the host. For example, the presence of a large number of B lymphocytes contribute to tuberculous granuloma formation through the release of chemokines and cytokines (7, 8). Granulomas can develop in any organ of the body where the causative infectious agent resides. They can also be formed in response to other persistent diseases such as; sarcodiosis schistosomiasis, syphilis, Crohn's disease, leprosy and in infections caused by bacteria, fungi and protozoa (9–18), and are either capable of containing the infection (latent TB), or not (active TB) (19–21). The rupture of a mature TB granuloma (the cause of which is not fully understood) in an individual with latent TB typically results in active TB (19, 21, 22). The most widely studied organs with TB granulomas are the lungs (6, 7, 19, 20, 23–28), whereas the characterization of TB granulomas in the brain, a site where infection with *Mtb* can have devastating consequences, is poorly described and understood (3). An improved understanding of TB granulomas in general could shed new light on improved approaches to TB management (20). There are, however, limited agreement on the conclusions to draw from studies of TB granulomas, such as lack of understanding of how the human granuloma functions. There is also no workable model that depicts the diseased human organ *in vitro*, as the structure and composition of granulomas vary among different organisms (6, 7, 29, 30). These are handicaps to our fuller understanding of TB. There is also no standard classification for the different types of granulomas identified to date. They are either grouped according to the composition of the immune cells, as primary or secondary granulomas (23, 29), or based on appearance and features such as: solid, caseous and cavitary, necrotizing, and non-necrotizing (5–7, 21, 22, 31). Consequently, the study of the TB granuloma needs to be expanded upon, using research methodologies, such as the ‘omics approaches, including, but not limited to, proteomics and metabolomics. Both proteomics and metabolomics have the potential of being instructive by elaborating upon the phenotype of TB granulomas at the protein and metabolite level, respectively.

This review provides a brief overview of TB; discusses the TB granuloma via function and importance, development, and categorization; and provides insights into the pro- and anti-inflammatory immune responses to *Mtb* by examining the limited proteomics studies (animal and human) conducted on TB granulomas and identifying specific metabolic indicators of TB progression and/or severity. The primary aim of this review is to indicate how proteomics and metabolomics have contributed, and can be expected to contribute, toward a better understanding of the TB granuloma extracted mainly from animal and human lung tissue. The secondary objective is to identify gaps in the literature that limit our understanding of the disease, in particular that there are few *in vitro* studies on TB granulomas in the brain. Lastly, inferences from current, as well as directions for future, ‘omics-related research on granulomas, are offered for tuberculous meningitis (TBM).

## Brief Overview of TB

Pulmonary TB begins with the transmission of the *Mtb* bacilli, via the transfer of *Mtb-*containing aerosols expelled from an infected person and inhaled by a non-infected person. TB affects the lungs more than any other organ of the body, since the lungs are usually the first point of entry into the host (32, 33). When these infected air droplets enter the new host, there is a subsequent activation of the host's innate agents conferring immunity, namely, neutrophils, dendritic cells and alveolar macrophages, which phagocytose the *Mtb* in the terminal alveoli (6, 34). The infected immune cells from the alveoli migrate to the lymphoid tissue, activating type 1 T-helper cells, producing pro-inflammatory cytokines such as interleukins (IL) and tumor necrosis factor alpha (TNF-α). These initial immune reactions lead to inflammatory changes in the lungs (30, 34–36). The Ghon focus, the primary site of infection in the lungs, enlarges as the disease progresses or the foci heal, leading to dense scars that may calcify (37). In an attempt to contain *Mtb*, macrophages engulf the bacilli and destroy them. However, if the process of elimination is unsuccessful, there will be continuous multiplication of the engulfed bacilli within the macrophage. Consequently, immune cells continue to work tirelessly in an attempt to contain the spread of the *Mtb*. In such instances, monocytes are subsequently recruited from the bloodstream to the site of infection, and the cycle of engulfment continues (32, 38). In a further attempt to neutralize the infection, the adaptive response/delayed type hypersensitivity is activated within 2–10 weeks after exposure to the infection (38, 39). This combined immune response to the invading bacilli eventually leads to the formation of a mature granuloma. Depending on the patient's immunity, the bacilli can be contained and the disease kept dormant (latent), not causing any clinical symptoms or disease or, typically in the case of individuals with a compromised immunity, active TB in which the infection spreads in the lungs. TB can progress from a latent form to an active state through the release of *Mtb* from the host's immune cells that have failed to contain/kill the bacilli (21). The progression from latent to active TB can be a result of several factors, such as immune deficiency, large or recurrent exposure to the disease, insufficient immune response to the pathogen, or exposure to a more virulent *Mtb* strain (6, 30, 40). Globally, about 23% of the human population have latent TB. The chances of individuals developing active TB from a latent infection are between 2–23%, and are higher (7–33%) in people living with human immunodeficiency viruses and acquired immunodeficiency syndrome (30, 41, 42). Latent TB can be detected only by carrying out tests such as the tuberculin skin test, interferon-gamma (IFN-γ) release assays, or the more recently developed QuantiFERON (Qiagen, Germany) and T.SPOT (Oxford Immunotec, UK) tests. Since these tests measure the resulting immune response *in vivo* or *in vitro* only, they cannot differentiate latent TB from resolved infection or active TB (43–45).

Of the 6.4 million TB cases reported by the World Health Organization (WHO) in 2017, 14% represent EPTB cases (1). EPTB occurs as a result of the lympho-hematogenous spread of bacilli to other organs of the body (46, 47). Once *Mtb* enters the host's immune system, the bacilli can replicate within the immune cells, thereby inducing the rapid production of chemokines and/or cytokines, which can be detected 2 h after infection (6, 7), and can be transported as far as the immune system carrying it travels (47). *Mtb* escapes the infected immune cells through the region of difference (RD1) locus (this locus is mostly found in virulent strains of *Mtb*), where the bacilli continue to replicate in the cytosol (38). The lymphatic endothelial cells, activated by IFN-γ, are said to be capable of restricting the RD1 locus of the bacilli from replicating (48), although this does not always occur sufficiently.

Tuberculous meningitis (TBM), the most lethal form of EPTB, is a form of CNS-TB where *Mtb* invades the meninges of the brain (49). Despite the protective properties of the blood–brain barrier (BBB) and the cerebrospinal fluid (CSF), *Mtb* are still able to enter the brain. The etiology of TBM is not fully known, but based upon research using animal models, it is proposed that *Mtb* enters the brain through infected immune cells, such as neutrophils and macrophages (the “Trojan horse” mechanism), and/or bind to endothelial cells of the brain (50–52). Once the bacilli penetrate the CNS barriers, they cause the resident immune cells (microglia) in the brain to be activated. Activation of microglia causes the release of numerous cytokines, which are important in the host's defense against *Mtb* infection, and which can also mediate inflammation. Microglia, rather than astrocytes, are preferentially infected by *Mtb*. The resulting neuroinflammatory processes lead to the disruption of the BBB, trigger the formation of vasogenic edema and recruit immune mediators that contribute toward BBB disintegration and the influx of the innate and adaptive immune cells from the periphery into the CNS (53, 54). The subsequent accumulation of these immune cells around the *Mtb* leads to the brain lesions typically seen in these patients. Rich and McCordock proposed that TBM is caused by the rupture of one of these lesions, the Rich focus; they are found in both the meninges and the brain parenchyma, and usually follow a vascular pattern (55–57). Activation of the Rich foci and the release of *Mtb* into the subarachnoid space, leads to additional cascades of devastating neuroinflammatory events (58).

During active *Mtb* infection, regardless of where it occurs in the body, there is a strong immune response involving clusters of differentiation of CD4+ and CD8+ lymphocytes and cytokines, including IFN-γ and TNF-α, produced by the dominant T-helper cell subset associated with the successful control of *Mtb* (59, 60). The inflammatory response subsequently causes IFN-γ to act in conjunction with TNF-α in order to activate macrophages and dendritic cells. IFN-γ and TNF-α are thought to play a crucial part in the complete eradication of *Mtb* because of their role in enabling the macrophages to produce reactive nitrogen intermediates and phagolysosome acidification (24, 61) to contain the bacilli. They do this by enhancing, stimulating, activating and mobilizing host defenses against the *Mtb*. These activated macrophages are essential for the formation of a granuloma (31). The pathogen is, however, merely contained (latent), maintaining a level of supposed inactivity, while the immune system constantly tries to maintain its defense with no clinical manifestations in the host (62–64). Despite this understanding of the initial mechanisms of *Mtb* infection, the perplexity of TB granulomas, in general, and especially so in the brain, still needs to be elucidated.

The function, importance and development of TB granulomas are comprehensively summarized in the next two sections, in order to provide a backdrop on our existing knowledge and a basis for future work.

## TB Granuloma Function and Importance

Granulomas are characterized by aggregates of lymphocytes, monocytes, plasma cells, mono-nuclear phagocytes, epithelioid cells, and giant cells – multi-nucleated fusion of immune cells, formed during chronic inflammation (29, 65, 66), formed in response to an infectious microorganism. The TB granuloma characterizes the host's immune response to an *Mtb* infection (67), and comprises an array of innate and adaptive immune cells, formed in response to signals given off by the infected macrophages, that trigger the migration of uninfected macrophages to the site of infection, in order to contain the *Mtb* invasion. However, in the case of failure to contain the bacilli, the TB granulomas can become shells in which the bacilli actually proliferate (6, 29, 65).

This poses the question: who benefits the most from the development of the granuloma – the host or the pathogen? The current precept backs the idea that a granuloma is a mark of an adequate and restrictive host immune response (68). However, studies (69–71) have shown that *Mtb* has the capacity to use the TB granuloma to its benefit. Upon initial infection, the bacilli recruit additional macrophages to contribute to the further spreading of the disease within the host. Macrophage infection triggers a localized pro-inflammatory response, resulting in the recruitment of activated innate immune components, including the neutrophils and dendritic cells. This then leads to the secretion of antimicrobial peptides (e.g., cathelicidin), cytokines (including IL-1α, IL-1β, TNF-α, IL-6 and IL-12), chemokines and additional macrophages that convene into a TB granuloma – a multicellular structure that cloisters the infecting *Mtb* from the surrounding tissue (36, 50, 72, 73). The formation of the TB granuloma is controlled by chemokines and cytokines, produced by local tissue cells and infiltrating leukocytes (74, 75). The organized, compact structure of the TB granuloma acts to constrain the *Mtb* infection and coordinates the activation of the immune cells, although the mechanism(s) surrounding this remains unclear (74). TB granulomas function via an interplay between the innate and cellular adaptive immunity (76), and orchestrate the interaction between immune cells, leading to an effective response to the infection: (1) the inhibition and killing of the *Mtb*; (2) containment of the infection and preventing the spread of the organism; or (3) localizing the inflammatory response and tissue damage (76, 77). TB granulomas can also serve as a niche for the invading *Mtb* (the bacilli can survive inside these structures for a relatively long time in a dormant state), while simultaneously protecting the host from active disease (78). Thus, TB granulomas have a duplicitous role. Owing to the dual role of the granuloma, the metabolism of *Mtb* and its survival within the host has been the subject of intense research. Because the bacilli depend completely on the host for survival, access to nutrients becomes a critical battle for survival (72). The *Mtb* genome subsequently evinces its metabolic flexibility and autonomy, as well as its capacity to resist nutrient stress (79). *Mtb* survives by producing sulfatides (which inhibit phagolysosomal fusion), cord factors (which inhibit neutrophil migration and damage mitochondria) and wax D (which controls intramural acidity, thereby making the DNA replication possible), which are all part of the *Mtb* cell wall (80–82). *Mtb* can persist and adapt to an *in vivo* environment by modifying gene expression; its metabolic capabilities enable the pathogen to cope in different stressful conditions within the host – (re)initiating the infectious cycle when environmental conditions are favorable (83, 84).

Changes in the number and the location of macrophages have an effect on the balance of TB granulomas and determine if the TB granuloma will effectively control the spread of the bacilli, or not (6). The structure of TB granulomas also changes as the disease progresses (85). These features are not yet completely understood and have given rise to several research studies using animal models; these, however, have not given a clear understanding of these phenomena in human granulomas (85–89). Furthermore, the TB granuloma studies performed in most animal species use other strains of mycobacteria, for instance, *Mycobacterium bovis* in sheep (91) and *Mycobacterium marinum* in zebrafish (90).

While there is much that we know about how the immune system responds to *Mtb*, much remains unknown. Within the tried-and-tested reductionist scientific approach, we can only answer the questions that we ask. What of the questions that we do not yet know how to ask? A new approach (‘omics) is needed to explore the dark side of the moon.

## Development of TB Granulomas

During the early infection phase, the interaction between macrophages and *Mtb* has a negative effect on the host – dampening the immune response and causing the increased survival of the *Mtb* bacilli (91). It has been proposed that the most abundant antigens of *Mtb* could benefit the pathogen rather than the host (92). The initial aggregation of macrophages occurs in response to a persistent stimulus triggered by a granulomatous reaction at its core (93, 94). Macrophages are distinct at the core of the granuloma, undergoing a series of distinguishable morphological changes exhibiting their immunometabolic characteristics, most importantly, epithelioid cell differentiation (94). Furthermore, macrophage polarization into M1 and M2 types is finely regulated by the host for the purpose of managing chronic infection(s), thereby regulating the promotion and formation of the granuloma (65, 94–97). A pro-inflammatory response, promoted by the M1 macrophages, bridges the innate and the adaptive immune response to infection. This functionality is considered the cornerstone of an effective host defense. At the same time, the M2 macrophages promote an anti-inflammatory response, which is crucial to immune regulation, also preventing an aggravated chronic inflammatory state, and simultaneously promoting the maintenance of tissue homeostasis (94, 95, 98). Lipid-laden macrophages, also known as foamy cells, are linked to TB granuloma necrosis, since they promote the formation of caseum – the build-up of necrotic debris at the core of the granuloma. When infected, foamy cells are packed with host lipids and are consumed by *Mtb* through aerobic glycolysis, thus promoting inflammation as well as transmission of live bacilli and the subsequent development of disease. Studies have shown localization and migration of *Mtb* toward the host lipid reservoirs (67, 99, 100), and the presence of foamy macrophages in TB granulomas (80, 81, 91, 92). Foamy macrophages seem to sustain intracellular *Mtb* in a physiological state, which explains the persistence and nutritional advantages to the *Mtb* bacilli (67, 101).

Natural killer (NK) cells, as well as B and T cells, dendritic cells, and neutrophils, are also recruited and contribute to TB granuloma function (76, 95). NK cells do not require antigen-specific recognition to kill target cells. IFN-γ, produced by CD4+, CD8+ and NK cells, and T-lymphocytes, are important mediators of macrophage activation, effector function and the immune response targeted against *Mtb* (76, 102). The dendritic cells and macrophages are mainly responsible for proteolytic processing and antigen presentation, and their functions include: regulating the overall immune response, initiating antigen-presenting cell activation, directing T-lymphocytes, activating NK cells, augmenting IFN-γ production, and directing antigen-induced cytotoxicity, (76, 103). The dendritic cells can also serve as a replication niche after ingesting *Mtb*, and are essential for antigen presentation to the T cells in the draining lymph nodes. However, pathogenic *Mtb* bacilli have developed mechanisms to prevent both dendritic cell migration and antigen presentation – an evolved adaptation of the pathogen (104, 105). In response to inflammatory stimuli, neutrophils are of the first cells to migrate to the infection site in response to the *Mtb*, in order to kill the pathogen, aided by the antimicrobial molecules (defensins, lactoferrin, cathelicidin, and lysozymes) contained in their granules (106–108). However, these immune reactions which target the pathogen may also lead to destruction of the surrounding host tissue, due to high levels of neutrophil influx, contributing to the development of a pathology serving as a “Trojan horse” for the *Mtb*. Although much is known about the immune cells that make up TB granuloma formation, much more remains to be learned about their functions in response to promoting or preventing disease (109–111).

The hallmark of the *Mtb* bacillus is a thick and waxy cell wall, whose unique design makes the bacilli difficult for the host to kill, which renders it rigid and impermeable, protecting the pathogen from dehydration and also rendering it resistant to conventional antibiotics (80, 84). Of the protective antigens of *Mtb*, mycolyltransferase and lipoarabinomannan are considered to be the most important. Modulating interactions with the immune system plays a key role in the infection cycle of *Mtb*, by blocking phagosome maturation and thus preventing lysis and antigen presentation, while also creating a favorable habitable environment for *Mtb* to replicate (112–115). Furthermore, as the immune system becomes activated, macrophages get stimulated with IFN-γ to increase proficiency against *Mtb* (83).

The *Mtb* serine/threonine protein kinase, called PknG, regulates bacterial metabolism as well as the pathogen's ability to survive inside the host by inhibiting phagosome maturation (116, 117). Studies show that PknG expedites *Mtb* growth and adaptation under *in vitro* stress conditions, such as nutrient deprivation, acid stress, and hypoxia (118–120). Under aerobic conditions, the bacilli are predicted to access glucose and triacylglycerides as sources of carbon throughout early replication; however, glucose-deficient macrophages trigger a metabolic shift in *Mtb* to utilize lipids (72, 121). PknG further disrupts host macrophage metabolic homeostasis to promote the accumulation of lipid bodies, complementing the aforementioned lipid use (122).

Hitherto, this review has summarized the basis of TB and its characteristic granuloma. From here on we examine several studies that illustrate the diverse categorization of TB granulomas, followed by the contributions offered by proteomics and metabolomics. The prime point of pretension proffered is that the categorization of TB granulomas is diverse.

## Categorization of TB Granulomas in Human or Animal Models

**Figure 1:**
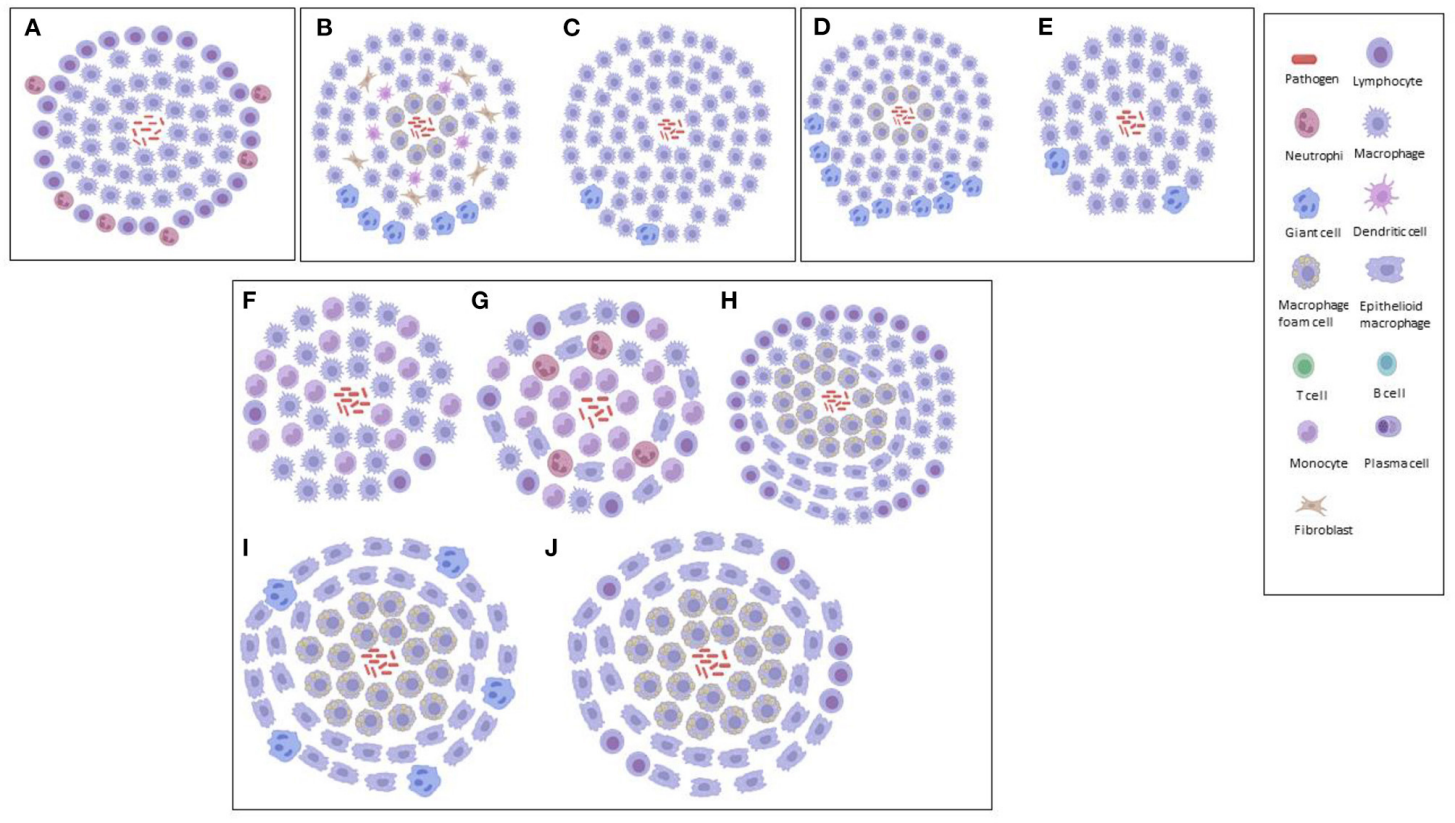
Graphical illustrations of TB granulomas based upon key cellular elements in their categorizations, based upon literature. **(A)** Foreign body-type and hypersensitivity granuloma. **(B)** Caseous and cavitary granuloma. **(C)** Solid granuloma. **(D)** Caseous and fibrocaseous granuloma. **(E)** Nasent granuloma. **(F)** Category 1 granuloma. **(G)** Category 2 granuloma. **(H)** Category 3 granuloma. **(I)** Category 4 granuloma. **(J)** Category 5 granuloma.

**Figure 2:**
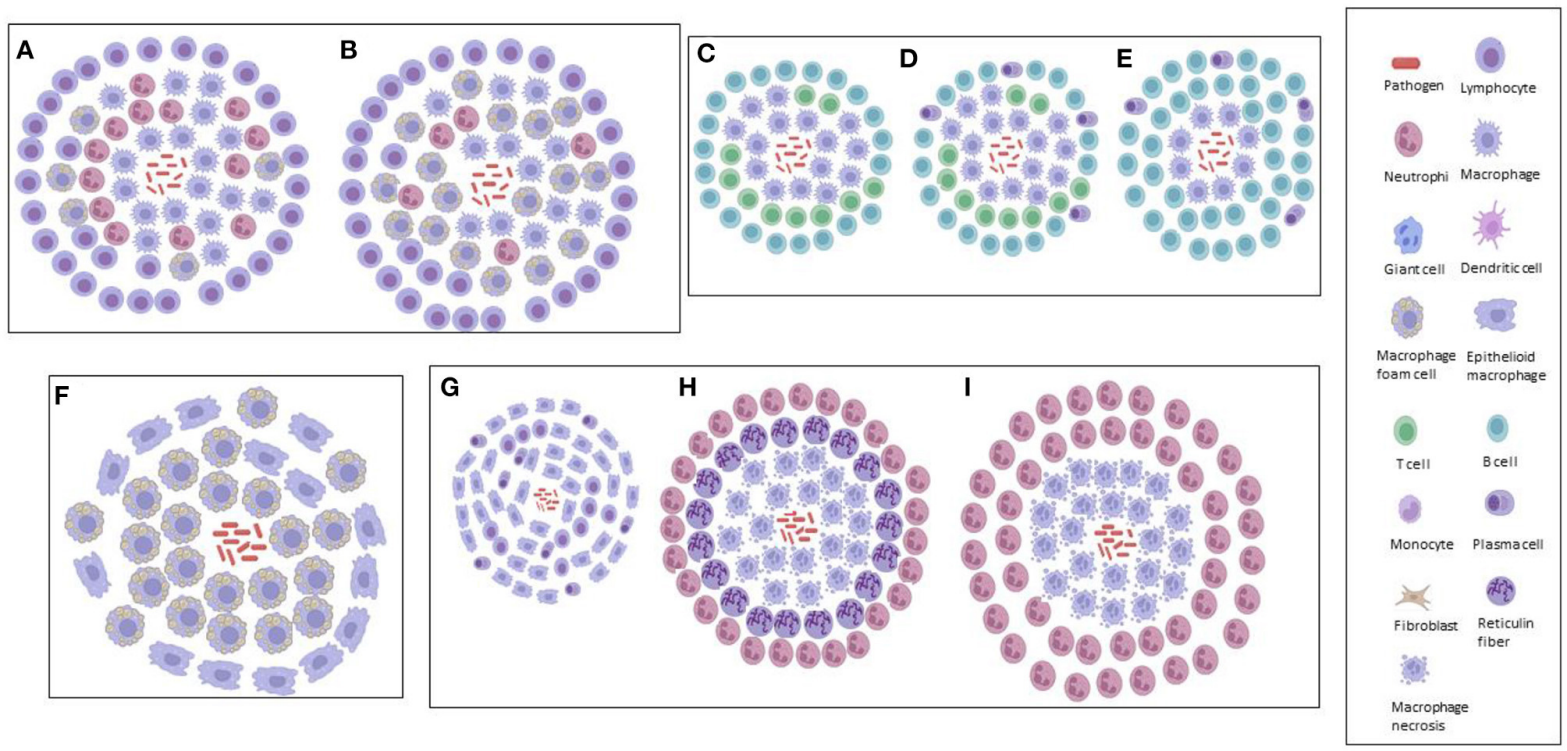
Graphical illustrations of TB granulomas based upon key cellular elements in their categorizations, based upon literature. **(A)** Primary granuloma. **(B)** Secondary granuloma. **(C)** Type 1 granuloma. **(D)** Type II granuloma. **(E)** Type III granuloma. **(F)** Early granuloma. **(G)** Non-necrotizing granuloma. **(H)** Necrotizing gummatous granuloma. **(I)** Necrotizing abscess granuloma.

A compact ordered aggregation of macrophages and their derivatives characterize granulomatous inflammation morphologically (40). It is thought that an understanding of these derivatives involved in inflammation (initiation and maintenance) could be important in assessing the development of the lesion, which in turn could help in the design of more effective and selective therapies (123). Subsequently, this has resulted in the categorization of the various TB granulomas, based on the varying derivatives associated with inflammation and/or *Mtb* interaction (Table 1). One key feature that distinguishes human TB granuloma from animal granuloma types is how they are structurally organized, although, differences are observed within granuloma types, rather than between the types. This could be as a result of the tissue location, cytokines, growth factors and associated cells, while the animal granuloma types may not be as organized as a result of the type of causative agent injected into the animal in order to induce immune responses similar to that observed in human TB granuloma (6, 20, 22). Figures 1, 2 illustrate our depictions of TB granulomas, based upon the information given on the categorizations below.

**Table 1:** Categorization of TB granulomas, based upon the literature.

**Type**	**Main findings**	**Mycobacteria strain**	**Site of granuloma(s)**	**Subjects**	**Reference**
Foreign body-type (non-immune) and hypersensitivity-type (immune) granulomas (Figure 1A)	The *Mtb* cell component trechalose 6,6 dimycolate is a pleiotropic molecule that induces both non-immune and immune TB granulomas.	*Mtb*	Lung	Mice	(40)
Solid, caseous and cavitary granulomas (Figures 1B,C)	Characterized by region of necrosis, TNF-α composition and presence of leukotriene A4 hydrolase (LTA4H): Solid – none. Caseous – high. Cavitary – low.	*Mtb*	Lung	Humans & rabbits	(5)
Nascent, caseous. fibrocaseous and resolved/calcified granulomas (Figures 1D,E)	Characterized based on the abundance of the proteins ADFP, ACSL1 and SapC: Nascent – low. Caseous – high. Fibrocaseous – high. Resolved – none.	*Mtb*	Lung	Humans	(21)
Categories 1–5 granulomas (Figures 1F–J)	Characterized based upon mononuclear phagocytes, alveolar macrophages and lymphocytes, and: 1 – few lesions. 2 – scattered, discrete foci. 3 – moderate-sized lesions. 4 – enlarged, coalescing lesions with small necrotic foci and advanced fibrosis. 5 – chronic, interstitial fibrosis of the lung and thickened granuloma wall.	*Mtb*	Lung	Mice	(124)
Primary, secondary and tertiary granulomas (Figures 2A,B)	Granuloma types explain disease severity in murine models: Primary (accumulated immune response) – beginning. Secondary (immunity acquisition) – middle. Tertiary (linkage with foamy macrophages) – late.	*Mtb*	Lung	Mice	(23)
Type I, type II and type III granulomas (Figures 2C–E)	Characterized by immune response routes: Type I (initial) – innate. Type II (developed) – adaptive cellular. Type III (terminal) – adaptive humoral.	*Mycobacterium bovis*	Bronchial lymph node	Sheep	(125)
Early granuloma (Figure 2F)	Early TB granuloma characterized by presence of mycobacteria and either epithelioid or foamy macrophages.	*Mycobacterium marinum*	Brain	Zebrafish	(90)
Non-necrotizing, necrotizing gummatous and necrotizing abscess granulomas (Figures 2G–I)	Characterized by size, reticulin fibers and *Mtb* load: Non-necrotizing – small, none and no *Mtb*. Necrotizing gummatous – medium, present and low *Mtb* load. Necrotizing abscess – large, none and high *Mtb* load.	*Mtb*	Brain	Humans	(31)

### Foreign Body- and Hypersensitivity-Type Granulomas

The pre-existing classification of lesions was according to their causative agents: infectious granulomas, foreign body granulomas and granulomas of unknown etiology (126, 127). Boros (128) revised this classification into the foreign body-type (non-immune) and hypersensitivity-type (immune) granuloma, in which classification was based on the involvement of antigen-specific lymphocytes in lesion development. The foreign body type was T-cell independent, whereas the hypersensitivity type was T-cell dependent. The hypersensitivity types were formed in response to foreign agents, which would require a delayed type of immunological response and would usually involve a high number of cellular responses. These agents could be bacteria, worms, fungi or viruses. The foreign body type were not formed in response to specific antigens and may be mostly inactive in the absence of specific foreign agents, such as a streptococcal cell wall, cord factor and silica (40, 123). Using a surface glycolipid derived from the cell walls of virulent strains of *Mtb –* trechalose 6,6 dimycolate (TDM) – Yamagami et al. (40) showed that TDM can induce both foreign body- and hypersensitivity-type granulomas in mouse lung tissue. Hence, both non-immune and immune mechanisms participate in granulomatous inflammation induced by *Mtb* infection.

### Solid, Caseous, and Cavitary Granulomas

Classification of granulomas by Marakalala et al. (5) was conducted on the basis of the histological characteristics of the *Mtb*-infected lung tissue collected from rabbits and humans. Solid granulomas lacked necrosis and had only one histologically distinct region. The caseous and cavitary granulomas had fused with an airway and both displayed two histologically distinct regions. The enzyme leukotriene A4 hydrolase (LTA4H) was present in abundance in caseous granulomas, less so in cavitary granulomas, and none was present in solid granulomas. LTA4H synthesizes leukotriene B4, a pro-inflammatory eicosanoid associated with the production of TNF-α, a key component of early host control of *Mtb* growth. From the protein and lipid snapshots of the human and rabbit lesions analyzed, Marakalala et al. (5) hypothesized that the pathological response to TB is shaped by the precise anatomical localization of these inflammatory pathways during the development of the granuloma.

### Nascent, Caseous, Fibrocaseous, and Resolved/Calcified Granulomas

Kim et al. (21) performed immunohistological analysis on lung tissues excised from TB patients. Tissues from 32 independent *Mtb*-infected samples (each sample had multiple granulomas) were categorized into four stages: nascent, caseous, fibrocaseous, and resolved. Their categorization of granulomas was based upon the proteins adipophilin (ADFP), acyl-CoA synthase long-chain family member 1 (ACSL1), and saposin C (SapC), as three key representatives of the lipid-modulating pathways that are impacted by the pro-inflammatory cytokine TNF-α. ADFP is a lipid droplet-associated protein present on the surface and in the core of intracellular lipid droplets formed in foam cells. ACSL1 mediates the formation of fatty acyl-CoA esters and a key component to stepin lipid biosynthesis and fatty acid degradation. SapC is involved in glycosphingolipid metabolism, and is also known to transfer mycobacterial lipid antigens, thereby activating the antigen-specific T cells. Nascent TB granulomas showed little-to-no presence of these three proteins, resolved/calcified TB granulomas showed none, and caseous and fibrocaseous TB granulomas demonstrated abundant ADFP, ACSL1 and SapC. The actual caseum of several TB granulomas revealed an abundance of ADFP, suggesting that the lipids of the caseum were likely to have been derived from the lipid droplets sequestered within foam cells upon the subsequent death of those cells. Kim et al. (21) further hypothesized that the TB granuloma undergoes a shift in lipid metabolism and accumulates host-derived lipids, supported further by the analysis of the lipid constituents of the caseous material isolated from human pulmonary TB granulomas. Hence, Kim et al. (21) proposed that granuloma formation was dependent on host lipid metabolism, mediated by the pathogen.

### Categories 1–5 Granulomas

Using mice aerogenically infected with *Mtb*, Rhoades et al. (124) categorized granulomas into five distinct immunopathological stages based upon the lesions formed in the lungs and their level of progress and development. Category 1 – small, isolated lesions composed of a few adjacent alveoli with thickened septae, scattered throughout infected lungs, consisting primarily of mono-nuclear phagocytes, alveolar macrophages and an occasional lymphocyte. Category 2 – scattered, discrete foci of alveolitis filled with mono-nuclear phagocytes and a few epithelioid macrophages, with perivascular and peribronchiolar lymphocytes. Category 3 – moderately sized granulomatous lesions characterized by sheets of epithelioid and foamy macrophages that fill the alveoli, with mild interstitial fibrosis and tight associations of lymphocytes. Category 4 – enlarged, coalescing granulomatous lesions consisting mainly of macrophages, including epithelioid cells, large foamy macrophages, and an occasional multinucleated giant cell, with small necrotic foci, advanced fibrosis of alveolar septae and a few remaining lymphocytes. Category 5 – late spectrum granuloma degeneration associated with chronic, interstitial fibrosis of the lung and characterized by thickened alveolar septae demarcated areas filled with dead/dying epithelioid and foamy macrophages. A major limitation observed by Rhoades et al. (124) was that the *Mtb*-infected mice did not develop caseating granulomas as is observed in humans. Rhoades et al. (124) further postulated that in animals the cytokine milieu around the lesions is different, perhaps leading to degeneration of the centers of large granulomas and necrotic caseation, and that the migratory capacity of the mouse lymphocytes is limited.

### Primary, Secondary, and Tertiary Granulomas

Also using the aerogenic infection model of mice with *Mtb*, Cardona et al. (23) simplified the histopathological categorization of TB granulomas in lungs as follows: primary, secondary and tertiary, based upon the immune reactions. A large accumulation of infected macrophages at the beginning of the infection defined primary granulomas, characterized by their large centers, and being surrounded by a lymphocytic mantle over time. After the acquisition of immunity against *Mtb*, secondary granulomas develop, in which scanty infected macrophages are surrounded by a thick lymphocytic mantle. Neighboring primary or secondary granulomas eventually become linked with foamy macrophages to form tertiary granulomas. Rhoades et al. (124) hypothesized a different histological evolution considering the migration of packed wedges of lymphocytes into granulomas, made by sheets of epithelioid and foamy macrophages, rather than foamy macrophages spreading out to the periphery of secondary granulomas (23).

### Type I, Type II, and Type III Granulomas

Using sheep infected with *Mycobacterium bovis*, Vallejo et al. (125) analyzed the bronchial lymph nodes for the immunohistochemical characterization of TB granulomas, based upon the number of acid-fast bacilli (AFB), T cells, B cells, macrophages and plasma cells each contained. Type I TB granulomas had <10 AFBs, no plasma cells, 1–10 T-lymphocytes, 11–50 B-lymphocytes and predominantly (51–100) macrophages. Type II TB granulomas had 10–20 AFBs, 11–50 plasma cells, 1–10 T-lymphocytes, 51–100 B-lymphocytes and predominantly (>100) macrophages. Type III TB granulomas had 10–20 AFBs, 51–100 plasma cells, no T-lymphocytes, >100 B-lymphocytes, and 51–100 macrophages that appeared peripherally. Thus, in the early/latent stages, an innate immune response would be more prominent. If the bacilli are not contained, there would be an increase in the number of macrophages that would subsequently attract more lymphocytes to the site of infection (adaptive cellular). Finally, a shift to a new phase in the immune response (adaptive humoral) would occur where B-lymphocytes and plasma cells would contain the infection with a humoral component (shift toward type 2 T-helper response) (125). Furthermore, Vallejo et al. (125) demonstrated how sheep are less than ideal models for studies using mycobacteria, since these animals were highly resistant, mostly attributed to their highly effective innate immune response that prevents the spread of the infection in the initial stages.

### Early Granuloma

Using an adapted zebrafish model of *Mycobacterium marinum* infection, van Leeuwen et al. (90) showed that the zebrafish–*M. marinum* model is particularly suitable for characterizing the early steps in the formation of brain granulomas, their immunological composition and the effect of bacterial virulence factors in the context of TBM, allowing for the detailed analysis of both bacterial and host factors involved in the disease. The brain is a notoriously difficult organ to analyse in terms of TB granulomas; hence, knowledge is limited. Van Leeuwen et al. (90) characterized the initial phases of zebrafish infection at the early developmental stages (innate immunity), with formation of bacterial clusters that were identified as early TB granulomas. All clusters contained both mycobacteria and a population of either epithelioid or foamy macrophages; however, their development was not influenced by changes to the BBB. The results of van Leeuwen et al. (90) suggest that traverse over the BBB occurs even at very low levels of extracellular bacterial loads, which led to their hypothesis that mycobacteria possibly make use of host cells – that is, macrophages – to migrate out of the bloodstream (which supports the concept of a “Trojan horse”).

### Non-necrotizing, Necrotizing Gummatous, and Necrotizing Abscess Granulomas

In one of the only studies to categorize TB granulomas in human brain tissue, Zaharie et al. (31) described three different types of granulomas – non-necrotizing, necrotizing gummatous and necrotizing abscess-type granulomas. The non-necrotizing granulomas are the smallest (0.1–0.5 mm) of the three, containing activated macrophages, lymphocytes and plasma cells, with absence of *Mtb* bacilli. The necrotizing gummatous granulomas (>5 mm) contain reticulin fibers in the center of necrosis and a relatively low *Mtb* bacilli load. The necrotizing abscess-type granulomas (up to 10 mm) are the largest, containing a large number of neutrophils, and completely absent of reticulin fibers in the center, with a high load of *Mtb* bacilli. Zaharie et al. (31) postulated that these different types of granuloma are part of the developmental stages of the pathological process. These authors (31) also challenge the classical theory of the Rich focus in TBM (58), by demonstrating that chronic granulomatous inflammation is initiated almost exclusively in the leptomeninges and not in the brain parenchyma, as opposed to the Rich hypothesis, in which the bacilli spread through the subarachnoid space, causing leptomeningitis after an active pulmonary infection.

All of the granuloma types reviewed here are categorized on the basis of the research interests of the researchers and writers. Perhaps if the granulomas are categorized according to disease severity, the organ from which the granulomas are extracted and characteristics of the cells that consist the granulomas, there could be a better understanding of how granulomas function and how such functions can be channeled to better understand the TB disease. The use of the ‘omics approach (discussed next) is a step in the right direction, which will give better insight, understanding and possibly help in the diagnosis of TB in the future. Also, more human post-mortem samples (not only from brain but from other organs where granulomas form) need to be studied in order to gain better insight into how the TB granuloma forms and functions as seen in the study conducted by Zaharie et al. (31).

### Challenges to TB Granuloma Categorization

Challenges to studying TB granulomas, specifically in the brain, include the lack of appropriate models and inaccessibility to human brain tissues, which has subsequently lead to the limited literature on the topic. Further limitations are due to the fact that direct inferences from the current animal models to humans cannot be made as conclusive outcomes of the disease since animals may have a different response to the disease and the mode of infection varies with that of humans; hence, the disease itself most likely presents differently. Therefore, there is still need to use human post-mortem tissue samples for the study of the human TB granulomas, as seen in the study of TBM in zebrafish in comparison with the study carried out on actual human post mortem samples, where notable differences were seen to exist between the granuloma developed in the zebrafish and that of human post-mortem samples (31, 90).

## Using Proteomics and Metabolomics to Understand TB Granulomas

The increasing demand and search for faster, more accurate methodologies that best describe the biochemical/pathophysiological status of granulomas have channeled efforts in the area of biomedical research recently to achieve better diagnostic and treatment methods (129, 130). From the need to augment work in biomedical research, the ‘omics era was born (131). The two such sub-disciplines that apply in the case of TB granulomas are proteomics and metabolomics.

### Proteomics

A proteome is loosely defined as all the proteins that can be expressed in a cell, tissue or in an organism, including the post-translational modified forms of proteins, and their isoforms (132). Proteomics – the study of the proteome – serves as a tool to advance biomedical research, such as vaccinology, drug discovery, and biomarker identification (133). The inherent focus of proteomics is to provide the most detailed insights into various cellular processes, by analyzing proteins, which cannot be captured by genomics or transcriptomics (134–136). Proteomics has come a long way over the last two decades and is currently in transition from its use in basic research to applications in medicine (134). During this period proteomics has contributed significantly to improving our understanding of the human pathogen *Mtb*, including application of improved intervention and prevention measures for TB, and providing exciting new insights into the adaptive life cycle of *Mtb* under different conditions, ranging from active metabolism and replication to dormancy (134, 135). Using proteomics, antigens expressed by *Mtb* under defined conditions with relevance for vaccine development have also been identified (137). Proteomics also enables superior characterization of proteins located in the *Mtb* cell wall, which previously were extremely difficult to identify (134, 138, 139). The application of proteomics has also contributed toward the understanding of TB granulomas (Table 2).

**Table 2:** Main findings from studies describing the role of proteomes and metabolomes in granulomas in both animal and human subjects infected with *Mtb*.

**Omics**	**Subjects**	**Site of granuloma(s)**	**Main finding**	**Reference**
**Proteomics**	Humans and rabbits	Lung	Inflammatory and antimicrobial effects of the center of the granuloma – known to be required to combat the *Mtb* infection but are destructive to host tissue, are contained by a ring of anti-inflammatory activity.	(5)
	Humans and mice	Lung and spleen	Novel *Mtb* functional antigens were identified, which were obtained directly from granulomatous lesions of TB patients	(140)
	Humans	Lung	Using immunohistochemistry, representative proteins were identified, which differed in their abundance levels in the caseous and cellular regions of granulomas.	(141)
**Metabolomics**	Guinea pigs	Lung	Metabolic changes seen are similar to the changes in the development of a tumor in cancer.	(85)
	Guinea pigs	Lung	Unique metabolic signatures were identified at different stages of the disease which may be useful for innovative and rapid diagnostic measures.	(142)
	Mice	Lung	*Mtb* infection triggers a temporary and progressive catabolic state to satisfy the continuous change in energy demand in order to control infection.	(143)

In a 2015 proteomics study, Marakalala et al. (5) combined laser-capture microdissection, confocal microscopy, and a Q-Exactive mass spectrometer coupled on-line to a nanoflow ultra-high-pressure liquid chromatograph (LC–MS–MS) to analyze over 3,000 proteins and a limited number of lipids during the different stages of TB granuloma formation in the lungs of humans and rabbits. Their main finding was that the center of the TB granuloma has a pro-inflammatory environment characterized by anti-microbial peptides, reactive oxygen species and pro-inflammatory eicosanoids, and that the tissue surrounding the caseum has an anti-inflammatory signature. Marakalala et al. (5) hypothesized that the pathological response to *Mtb* is shaped by the precise localization of these inflammatory pathways during the development of the granuloma. Hence, the organization of the TB granulomas promotes antimicrobial activity while limiting host tissue destruction. These results were consistent across both human and rabbit lung tissue samples.

Also in 2015, Yu et al. (140) used free-solution isoelectric focusing to obtain chaperone-rich cell lysates from the granulomatous lung lesions of active TB patients, and combined the technique with high-resolution orbitrap mass spectrometry to identify six *Mtb*-associated peptides not noted in the control samples. They further identified a peptide (PKAp) derived from *Mtb* protein kinase, which not only contributed to significant antigen-specific IFN secretion, but also to cytotoxic lymphocyte function and T-cell proliferation. Yu et al. further validated these results *in vivo* with mice immunized by the PKAp peptide, showing increased cellular IFN-γ in both the lungs and spleen, without causing immunopathogenesis/disease. Mice immunized with the PKAp peptide alone showed increased IFN- γ secretion only in lung CD4+ T cells but not in spleen cells (140).

A recent proteomics study (2020) by Seto et al. (141), applied mass spectrometry-based proteomics combined with laser microdissection, in order to investigate the unique protein markers in human mycobacterial granulomatous lesions. These researchers compared protein abundance (2,812 proteins) in the caseous regions between TB and *Mycobacterium avium* complex lung disease (MAC-LD) granulomas and demonstrated the expression of mycobacterial proteins by proteomic profiling, and showed a significant change in the abundance of several proteins in MAC-LD caseum relative to those of the TB caseum. Enrichment analysis further revealed that neutrophil proteins were accumulating in the caseum region. Proteins in the proteasome were abundant in the TB cell region, suggesting that antigen processing and presentation by dendritic cells and macrophages actively occur in this region (i.e., active innate immune response).

Considering the above, proteomics has shown that TB granulomas observed in human lungs have specific protein markers unique to each granuloma type; TB granulomas are different in the composition and abundance of cells that are part of the immune response; features depend on the causative agent of the disease (5, 78, 140, 141).

### Metabolomics

Metabolomics is capable of quantifying all low molecular weight compounds (metabolites; collectively known as the metabolome) in biofluids, cells, tissues, and whole organisms, and is designed to quantitatively analyze and describe molecular phenotypes (144). Metabolomics is an omics technique that has been adapted for the study of infectious diseases – broadening our knowledge with respect to its use for prognostic and diagnostic purposes, specifically the screening of disease-specific biomarkers (145). Metabolomics has been, and is still being, used to study the biological mechanisms of TB both *in vitro* and *in vivo*, in animal models and human patients (146–148). The technique has revealed the carbon sources available to pathogenic *Mtb in vivo* (149, 150), and has led to the identification of metabolic profiles associated with TB in animal models. It has also been used to characterize human latent and active TB infection by means of NMR-based analysis of serum, as well as MS-based analysis of sputum (151–154). These studies have been reviewed by Du Preez and Luies (146) and Du Preez and Loots (153). Furthermore, metabolomics has also served as an effective research tool for the identification of specific novel TB metabolic markers (155, 156) from patients' sputum, plasma, serum and urine (151, 157–159). However, all of these metabolic markers identified to date are only associated with biofluids, with very few instances where the actual infected tissue (in particular lungs or brain) were analyzed. Hence, there is still a need to isolate the infected tissue (TB granulomas) and study the altered host and *Mtb* metabolism at the site(s) of infection.

Considering the latter, an *ex vivo* study using untargeted high-resolution magic angle spinning nuclear magnetic resonance (NMR) spectroscopy was carried out on lung tissue of guinea pigs infected with *Mtb* (85). The study showed significantly elevated lactate, alanine, acetate and glutamate, aspartate, creatine, phosphocholine, glycerophosphocholine, betaine, trimethylamine N-oxide (TMAO), myo-inositol, scyllo-inositol, and dihydroxyacetone, as well as reduced phosphatidylcholine, and oxidized and reduced forms of glutathione. These altered metabolites indicate utilization of alternate energy sources by the infiltrating cells that generate much of the metabolites in the increasingly necrotic and hypoxic developing granuloma, via the glycolytic, pentose phosphate, and tricarboxylic acid pathways. Furthermore, the metabolic changes seen in their study were very similar to what was previously reported in cancer during tumor development. Somashekar et al. (85) concluded that their non-destructive metabolomics approach is particularly relevant in studying the metabolic fate of *Mtb* during human infection and that metabolic fingerprints reflect the cycle of active replication and persistence of *Mtb* during granuloma formation in all stages of infection. In 2012, Somashekar et al. (143), again using NMR metabolomics, analyzed lung tissue collected from guinea pigs infected with the W-Beijing *Mtb* strain, and indicated a characteristic metabolic difference (in respect of acetate, alanine, glutamate, aspartate, glutathione, phosphocholine, creatine, glutathione, glycerophosphocholine, betaine, myo-inositol, TMAO, dihydroxyacetone, and scyllo-inositol) compared to the control group, which also changed as the disease state progressed. This metabolic profile closely matched that observed in their previous study (85). However, in this investigation they additionally observed an increase in glutathione in response to elevated oxygen radicals produced in the lesions. These researchers also indicated that use of NMR in this study can be used to identify changes to important pathways that occurred as a result of disease progression, and that these metabolic signatures could possibly be used in the development of improved vaccination or therapeutic strategies.

In a study on mice, using high-resolution mass spectrometry coupled with the powerful imaging technology of MALDI, Prideaux et al. (160) indicated that *Mtb* uses TMAO as an electron acceptor under anaerobic conditions. They additionally reported that high abundances of TMAO are produced by the host, in response to the elevated concentrations of trimethylamine, a substrate supplied by the *Mtb* after infection. They concluded that the lung tissue metabolome can be greatly altered as TB disease progresses, and untargeted metabolomics is an extremely valuable approach to understand better those metabolic changes associated with site-specific *Mtb* infection (142).

Considering the above, metabolomics can be expected to be useful in advancing our understanding of granuloma formation in TB, and in determining the unique metabolic signatures of different granuloma and tissue types (such as the brain). Of particular interest, in all three of the metabolomics studies on TB granulomas described above, TMAO was a common metabolite and so worthy of further investigation.

## Mind the Gap

Table 1 shows that TB granulomas manifest great diversity, depending on the source material and whether it is associated with inflammation caused by *Mtb* infection. The table also reveals that our knowledge of these granulomas is predominantly based upon examination of lungs, in both human and animal studies. Only two investigations have reported EPTB granulomas – in the bronchial lymph nodes in sheep (125) and the brain in zebrafish (90). Just one study, by Zaharie et al. in 2020, has examined TB granulomas in the human brain (31). This work, which was based upon immunohistochemistry analyses and three-dimensional computer modeling, challenges the classical theory of the Rich focus (58) in TBM. Zaharie et al. demonstrated that most of the chronic granulomatous inflammation was located in the subarachnoid space (leptomeninges) and to a much lesser extent in the superficial brain parenchyma (adjacent to the pia mater). They hypothesized that the superficial intraparenchymal component is just a transpial or perivascular extension (Virchow-Robin spaces) of a leptomeningeal granuloma. Furthermore, all three granuloma types studied by Zaharie et al. showed no observable differences between adult and children, highlighting that the immune response in a developing CNS that has been impacted by an infection functions in a similar manner to that of a developed CNS; however, hydrocephalus – a physiological complication of infection, occurs in more frequency in children (31). Thus, it is very clear that we need more research on the TB granulomas in the brain, especially in children, in order to understand the immunopathogenesis of TBM – one of the major knowledge gaps identified by the US National Institutes of Health (161) at a 2018 workshop.

The advantage offered by untargeted proteomics and metabolomics is that the exploratory nature of their analyses examines the research subject with an open mind and in a holistic fashion, and permits hypotheses to be generated based upon discoveries. This non-biased research approach is ideal for providing new biological insight(s) that are otherwise difficult to achieve via the traditional reductionist scientific method. Discoveries that have been elucidated are that the metabolite TMAO plays an important role in the lung granuloma of animal models, and that the localization of specific proteins in granulomatous lesions in the lungs of human TB patients are distributed specifically to counter *Mtb* infection, while containing the contents of the granuloma in order to limit tissue damage. The sustained to-and-fro battle between host and *Mtb* is immensely complicated, which is reflected in the complexity of the TB granuloma. This complexity is compounded by the biological variability among individuals; hence, each host responds differently to the assault by the pathogen.

Although our data on the role of TB granulomas in the human brain are extremely limited, we predict that applying proteomics and metabolomics to the study of TB granulomas in the brains of human TBM cases should provide new insights into the pathogenesis of the disease. This newfound understanding could then be translated into practical application toward reducing the severity of this devastating, often fatal, neurology.

## Concluding Remarks

The WHO aims to eradicate TB completely by the year 2030 (162) – an ambitious task. In order to achieve this, it is necessary to identify those living with latent TB, and to neutralize the threat before they become infectious (45). Identifying specific markers peculiar to patients with latent TB would be beneficial to tackling this challenge. The use of bioinformatics, and the other ‘omics' disciplines, have proved able to identify several potential targets for the survival of *Mtb* in the latently infected host (163–165).

Proteomics and metabolomics studies, using human and the increasing sophistication of animal modeling have generated new ideas about the roles of TB granulomas; nonetheless, many questions still remain unanswered regarding the host's response to TB. For example, from work on the immunology and immunometabolic events in TB granulomas (166), two major points for future research have been identified, namely: (1) What is the nature and function of the differential spatial organization of immune cells within the TB granuloma and how does the expression of the different anti-TB enzyme systems change?; and (2) as T cells are essential to constrain the spread of infection, what are the metabolic cues during TB-specific T-cell trafficking between TB granulomas and draining lymph nodes? Nevertheless, more multidisciplinary ‘omics studies, such as gene expression studies (167), especially in human subjects, are required to contribute toward ushering in a new era of understanding of TB granulomas – both at the site of infection, and on a systemic level.

## Author Contributions

SM conceived the review. AS, AW, DL, and SM planned the outline and structure. AS wrote the manuscript. AW, AT, DL, MK, and SM read various drafts of the manuscript and contributed and incorporated by AS. All authors read and approved of the final version of the manuscript.

## Conflict of Interest

The authors declare that the research was conducted in the absence of any commercial or financial relationships that could be construed as a potential conflict of interest.

## Publisher's Note

All claims expressed in this article are solely those of the authors and do not necessarily represent those of their affiliated organizations, or those of the publisher, the editors and the reviewers. Any product that may be evaluated in this article, or claim that may be made by its manufacturer, is not guaranteed or endorsed by the publisher.
